# Guarding against digestive-system cancers: Unveiling the role of Chk2 as a potential therapeutic target

**DOI:** 10.1016/j.gendis.2023.101191

**Published:** 2023-12-07

**Authors:** Yucheng An, Duolun Gao, Yanjie He, Nan Ge, Jintao Guo, Siyu Sun, Caixia Wang, Fan Yang

**Affiliations:** a Department of Gastroenterology, Shengjing Hospital of China Medical University, Shenyang, Liaoning 110004, China; b Department of Surgery, New York University School of Medicine and NYU-Langone Medical Center, New York, NY 10016, USA

**Keywords:** Checkpoint kinase 2, Cell-cycle arrest, Digestive-system cancer, Radiochemotherapy resistance, Tumorigenesis

## Abstract

Digestive-system cancers represent major threats to human health; however, the mechanisms underlying tumorigenesis and radiochemotherapy resistance have remained elusive. Therefore, an urgent need exists for identifying key drivers of digestive system tumorigenesis and novel targeted therapeutics. The checkpoint kinase 2 (Chk2) regulates cell-cycle progression, and Chk2 dysregulation or Chk2 mutations can lead to the development of various cancers, which makes Chk2 an important research topic. This review summarizes the roles of Chk2 in DNA-damage responses, cell-cycle regulation, autophagy, and homeostasis maintenance. We describe relationships between tumorigenesis and cell-cycle dysregulation induced by Chk2 mutations. In addition, we summarize evidence indicating that Chk2 can serve as a novel therapeutic target, based on its contributions to radiochemotherapy-resistance reversion and progress made in developing antitumor agents against Chk2. The prevailing evidence supports the conclusion that further research on Chk2 will provide a deeper understanding of digestive-system tumorigenesis and should suggest novel therapeutic targets.

## Introduction

Digestive-system cancers, *i.e.*, gastric cancer (GC), colorectal cancer (CRC), liver cancer, and esophageal cancer (ESC), which are among the top 10 most commonly diagnosed tumors, have proven to pose great threats to human health and safety globally. Together, GC, CRC, and hepatic cancer account for 20.3% of the total morbidity of all non-cutaneous cancers.^1^ Among them, CRC has the third highest incidence rate of all tumors, which has increased 2- to 4-fold over the last two decades in many developing countries.^2^ GC is second leading cause of cancer-related death, and over 1 million cases of GC occurred globally in 2020, with over half of all cases occurring in China.^1^^,^^3^ Although different anti-tumor therapies (including surgical resection, radiochemotherapy, and targeted drugs) have greatly helped patients overcome GC, the 5-year survival rate is only 5%–8% in patients with advanced digestive cancers.^4^^,^^5^ Thus, more research is needed to explore key tumorigenic factors and develop more effective therapies.

The checkpoint kinase 2 (Chk2) gene is located on chromosome 22q12.1 and contains 14 exons that stretches from base pair 28,687,742 to base pair 28,741,904. The Chk2 protein, encoded by the Chk2 gene, is a serine-threonine kinase consisting of 543 amino acids that regulates cell-cycle progression and exhibits anti-cancer activity. The N-terminal region of the encoded protein contains a domain rich in serine–glutamine and threonine–glutamine pairs, which is known as the SQ/TQ cluster domain. The SQ/TQ motifs are phosphorylated by members of the PI3K kinase family, including ATM and ATR. Like many protein kinases, the catalytic function of Chk2 kinase is activated by phosphorylation of a peptide region located in the kinase domain but outside the active site cleft (called the activation loop or the T-loop; residues 366–406). The T-loop contains several residues that are autophosphorylated, which leads to effective kinase activity. During normal growth, Chk2 exists in an inactive, monomeric form in the cell nucleus. However, ionizing radiation or other external factors that cause DNA damage can activate Chk2 via phosphorylation of threonine 68 in an ATM-dependent pathway.^6^, ^7^, ^8^

Tumors comprise a class of diseases involving disrupted cell cycle-regulation mechanisms, and sites important for cell-cycle progression play central roles in the overall monitoring system of cell cycle processes. Chk2 is a very important protein kinase that functions at cell-cycle checkpoints and regulates the activities and expression of downstream target proteins through signaling and amplification to cause cell-cycle arrest and regulate tumor development. Chemotherapy resistance is a major factor that leads to tumor relapse, increased difficulty in treatment, treatment failure, and a poor prognosis for patients with cancer. The DNA-damage response (DDR) pathway affects chemotherapy resistance in tumors. Targeting DDR components such as DNA-repair kinases or checkpoint regulators can provide new treatment solutions. In recent years, the Chk2 protein (which regulates the DDR process) has attracted widespread attention from scholars because it is thought to trigger mitotic catastrophe and thus promote cell death, suggesting it is a promising target for cancer therapy. Chk inhibitors have been used in clinical practice and have achieved some success in reversing acquired drug resistance caused by cell-cycle dysregulation by inducing apoptosis and causing cell-cycle arrest.

**Figure 1:**
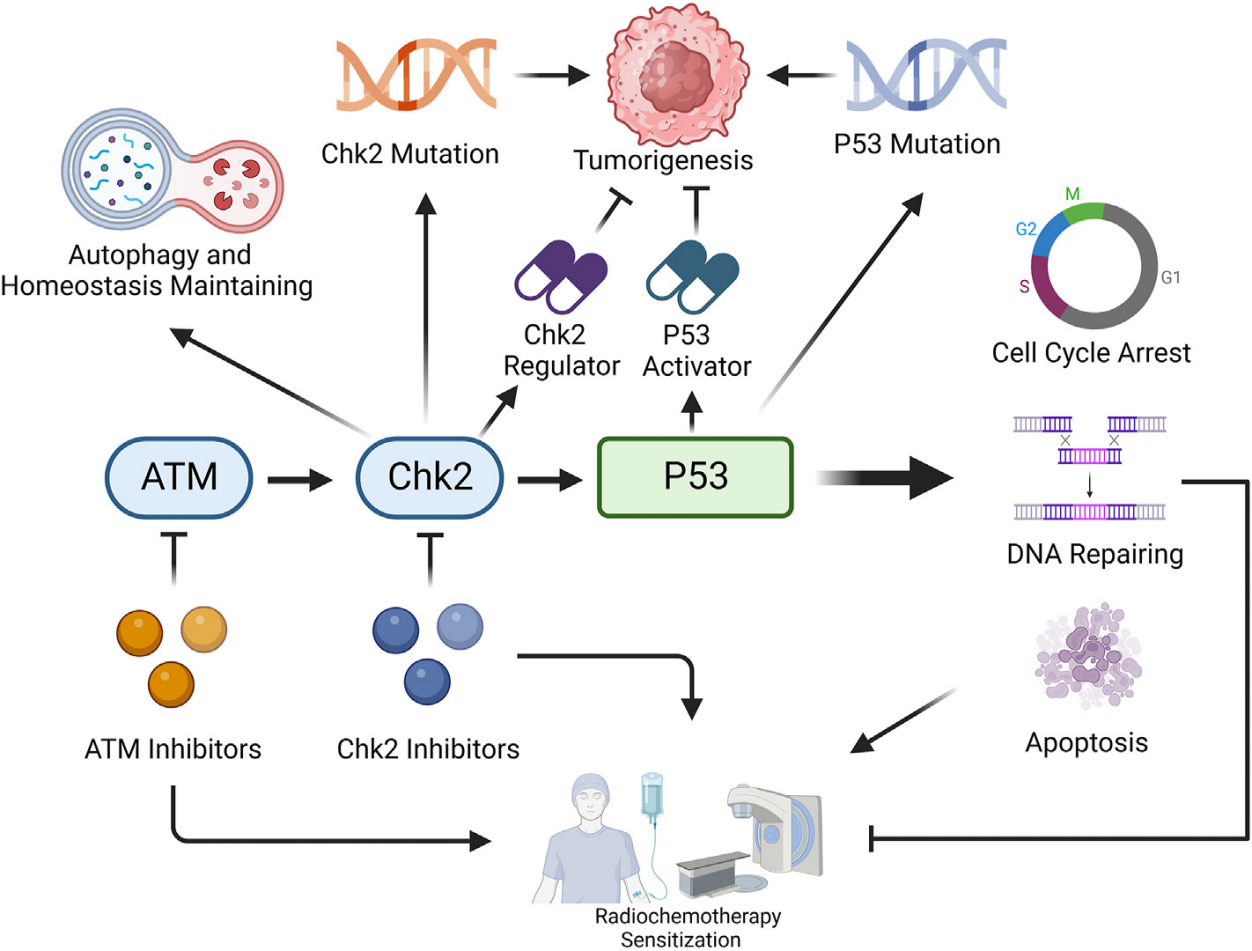
ATM-Chk2-P53 pathway as a target for anti-tumor treatments. ATM-Chk2-P53 pathway is critical in cell cycle arrest, DNA repairing, apoptosis, and tumorigenesis. Mutations on Chk2 or P53 may induce tumorigenesis, meanwhile, both of them are potent targets for anti-tumor agent development. Chk2 regulators and P53 activators have been proven to suppress tumorigenesis. Effective DNA repairing contributes to radiochemotherapy resistance while apoptosis leads to radiochemotherapy sensitization. ATM and Chk2 inhibitors are both potent sensitizers for radiochemotherapy, however, more clinical trials will be needed before they are applied clinically.

In this review, we summarize progress made in understanding the role of Chk2 in digestive-system tumors. We cover the basic function of Chk2, its tumorigenic mechanism, and how Chk2 signaling relates to chemoradiotherapy resistance. We also summarize the contributions of Chk2 inhibitors and drugs that target Chk2 pathways to anti-tumor therapy. It is worth mentioning that we also summarize the novel roles of Chk2 in autophagy and homeostasis maintenance. We hope that this updated review will be enlightening for researchers conducting digestive-cancer studies (Fig. 1).

## Chk2 and its role in cell survival

### Classical roles of Chk2 in the DDR pathway and cell-cycle arrest

Because of various endogenous or exogenous DNA-damaging agents, human cells are subjected to DNA damage every day.^9^ Hydrolytic and oxidative reactions induce DNA-base lesions every day, and products of macrophages and neutrophils attack DNA leading to base losses or single-strand breaks. DNA double-strand breaks occur when two single-strand breaks are close to each other, when single-strand breaks are encountered during DNA replication, or when exogenous DNA-damaging agents are present.^10^ The accumulation of lesions and genome aberrations can lead to cell death or cancer phenotypes if the damage cannot be repaired in time.^11^ The DDR pathway involves a series of reactions including lesion sensing, which generate appropriate signals and promote DNA repair. In the DDR pathway, proteins recognize lesions and activate upstream DDR kinase, mediator proteins that facilitate phosphorylation events, and downstream effectors, such as proteins responsible for DNA repair and cell-cycle arrest.^12^^,^^13^

### Cell-cycle arrest

The human cell cycle consists of four main phases: G1, S, and G2 of interphase, and M phase of cytokinesis. Cells repeat this process regularly, and cell-cycle checkpoints regulate the process in real time. Chk2 serves as a cell-cycle checkpoint that coordinates cell-cycle progression with repair pathways, transiently stopping cell division or delaying cell-cycle progression to provide sufficient time to repair damage during the critical phases of DNA replication and mitosis.

When a double-strand break occurs, the MRE11–RAD50–NBS1 complex recruits ATM to the lesion site as a sensor and promotes ATM activation through autophosphorylation.^14^ Then, downstream factors (such as Chk1 and Chk2) are recruited and activated, which induces cell-cycle arrest and apoptosis.^15^ ATM phosphorylates Chk2 at threonine 68 and initiates downstream effectors. Chk2 can mediate cell-cycle arrest through the following main mechanisms. ATM and Chk2 up-regulate P53 phosphorylation (and thus activation), followed by P21 up-regulation (an inhibitor of cyclin-dependent kinases). Subsequently, P21 overexpression induces G1/S phase arrest. In addition, Chk2 phosphorylates and thereby inactivates Cdc25A, which prevents CDK2 activation and promotes G1/S phase arrest. G2/M phase arrest is induced by the abnormal activation of the cyclin B1–cyclin-dependent kinase 1 (CDK1) complex, which is essential for the G2/M phase transition. Chk2 phosphorylates Cdc25C, which causes Cdc25C to translocate to the cytoplasm. Cdc25C cannot activate the cyclin B1–CDK1 complex in the cytoplasm, which further promotes G2/M phase arrest.^6^^,^^16^^,^^17^

DNA repair mainly occurs through nonhomologous recombination and homologous recombination. Nonhomologous recombination is more effective with the human genome, although homologous recombination is more common overall in eukaryotic cells, perhaps because homologous recombination is more accurate.^18^^,^^19^ Chk2 mainly participates in the homologous recombination mechanism by phosphorylating breast cancer-susceptibility proteins 1 and 2 (BRCA1/2), after which phosphorylated BRCA1 recruits the recombinase Rad51 and phosphorylated BRCA2 disrupts the Rad51–BRCA2 complex and enables Rad51 to participate in homologous recombination.^20^^,^^21^

Chk2 can also participate in cell-cycle arrest through other pathways. For example, Chk2 can phosphorylate the Rb protein, which enhances the formation of the transcriptionally inactive pRb–E2F-1 complex, thereby leading to G1/S arrest and apoptosis suppression. Additionally, Chk2 phosphorylation may induce the serine/threonine protein kinase LATS2, which can cause G1/S arrest.^22^ Researches have also proposed three other mechanisms that lead to cell-cycle arrest at the G2/M phase. One mechanism involves phosphorylation of the cell death-resistant transcription factor, Che-1, by both Chk2 and ATM, leading to Che-1 stabilization, Che-1 recruitment to the p21 and p53 promoters after damage, subsequent p21 and p53 transcriptional activation, and the maintenance of cell-cycle arrest at the G2/M phase.^23^ Another mechanism involves Chk-2-dependent phosphorylation of a p53 co-factor (the serine/threonine kinase receptor-associated protein, STRAP) by ATM, which promotes STRAP nuclear localization, enhances p53 stability, and induces p53-dependent G2/M phase arrest. The third mechanism involves the Chk2-mediated phosphorylation and stabilization of the dual-specificity protein kinase TTK/hMps1, which leads to G2/M arrest.^24^, ^25^, ^26^

### Apoptosis induction by DNA-repair failure

**Figure 2:**
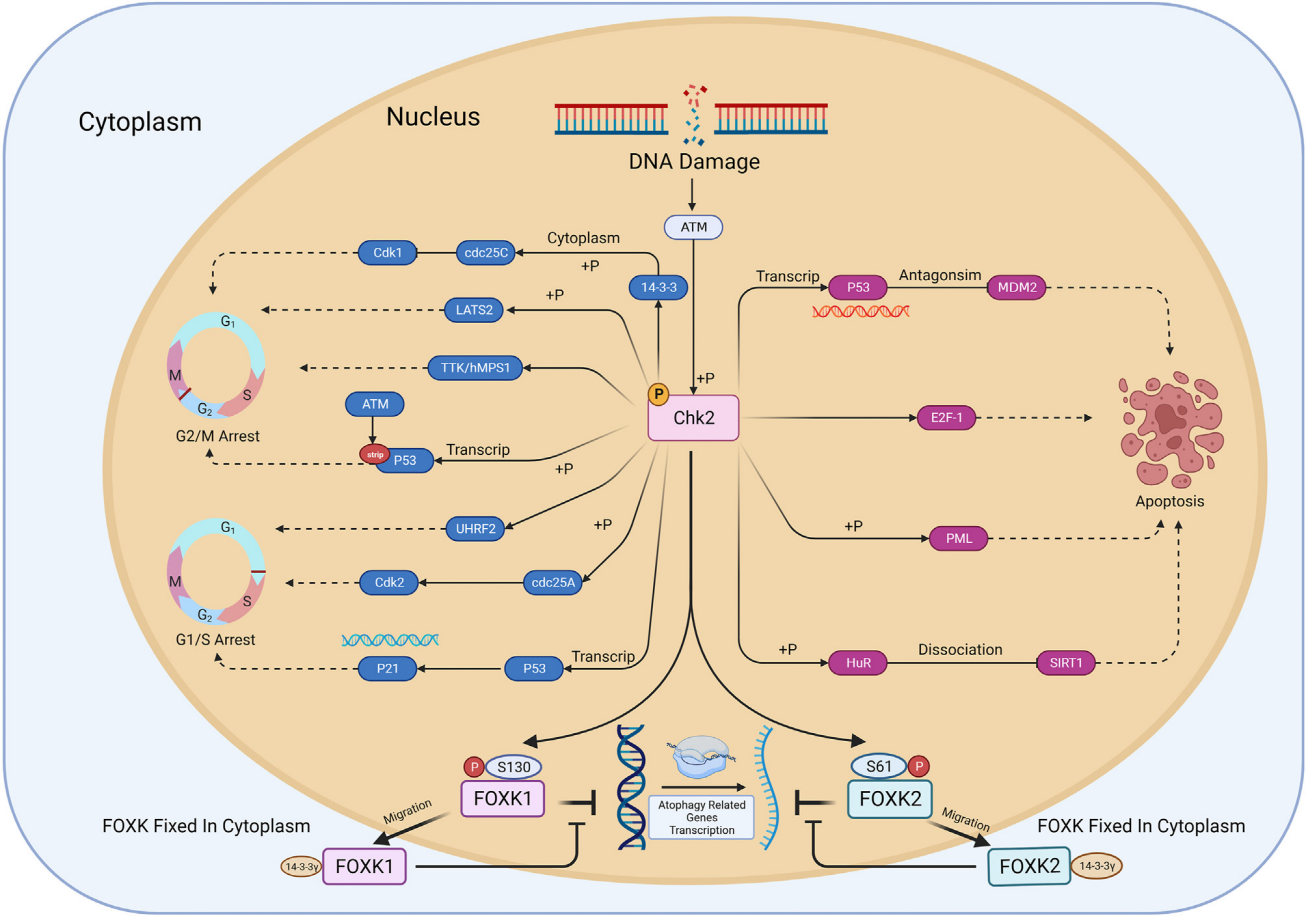
CHK2 in cell cycle checkpoint activation. Chk2 phosphorylates cdc25C and translocates it into the cytoplasm via 14-3-3 protein, which inhibits Cdk1, leading to G2/M arrest. Phosphorylated LATS2 and TTK/hMPS1 by CHK2 can promote G2/M arrest. CHK2 phosphorylates P53 and ATM phosphorylates STRIP, inducing the expression of the P53 gene and promoting P53-dependent G2/M arrest. In addition, CHK2 promotes G1/S arrest by phosphorylating UHRF2. It also phosphorylates cdc25A, activating Cdk2 and inducing G1/S arrest. Furthermore, CHK2 phosphorylates P53, inducing P21 expression, and leading to G1/S arrest. When DNA damage cannot be repaired, CHK2 phosphorylates P53 and promotes P53 accumulation, antagonizing MDM2 and inducing P53-dependent cell apoptosis. Additionally, CHK2 can induce P53-independent apoptosis by targeting E2F-1 and phosphorylating PML. Moreover, CHK2 can phosphorylate HuR, promoting the dissociation of HuR and SIRT1, reducing the abundance of SIRT1, and regulating cell apoptosis. Furthermore, DNA damage in the nucleus recruits ATM to phosphorylate Chk2, subsequently inducing the phosphorylation of FOXK1 at the S130 site and FOXK2 at the S61 site. The phosphorylated FOXK protein then translocates from the nucleus to the cytoplasm, where it is sequestered by binding to 14-3-3γ. Cytoplasmic FOXK, released from its transcriptional repression on autophagy related genes, promotes autophagy.

When DNA damage cannot be repaired, the Chk2 kinase can initiate apoptosis. The results of numerous studies have shown that Chk2 is essential for regulating the p53-dependent cell-death pathway. Both endogenous and exogenous apoptosis require the participation of p53. In non-stressed cells, p53 has low activity and a short half-life, mainly because p53 forms complexes with two proteins, *i.e.*, E3 ubiquitin-protein ligase (Mdm2) and MDM2-like p53 binding protein, which leads to p53 ubiquitination and proteosomal degradation and causes cells to grow normally. After DNA damage, ATM and Chk2 can become activated, which in turn activates p53 via phosphorylation on Ser15 and Ser20, thus antagonizing MDM2 and promoting nuclear p53 accumulation and apoptosis.^27^ Chk2 can also support p53-independent apoptosis by phosphorylating the transcription factor E2F-1 at Ser364, leading to stabilization of the transcription factor and, consequently, transcriptional activation and apoptosis induction in a p53-independent manner. In addition, Chk2 can phosphorylate the tumor suppressor gene PML at Ser117, which increases p53-independent pro-apoptotic activity. Moreover, Chk2 phosphorylates Hu-antigen R at residues Ser88, Ser100, and Thr118, and then Hu-antigen R dissociates from NAD-dependent protein deacetylase sirtuin 1, which lowers the expression of sirtuin 1 and promotes apoptosis. Interestingly, in wild-type HCT16 CRC cells, Chk2 caused almost all Hu-antigen R–mRNA complexes to dissociate, and several Hu-antigen R-interacting mRNAs that encode apoptosis and proliferation-related proteins (TJP1, Mdm2, TP53BP2, Bax, and K-Ras) dissociated from Hu-antigen R, which enhanced cell survival^28^, ^29^, ^30^ (Fig. 2).

### Novel roles of Chk2 in autophagy and homeostasis

Cellular homeostasis is vital for normal metabolism and biological functions in biological organisms. During evolution, cells acquired physiological functions such as the DDR pathway, cell-cycle arrest, cell senescence, autophagy, and apoptosis, which are responsible for maintaining cell homeostasis. Autophagy is a self-degradative process that eliminates specific proteins, injured organelles, and intracellular pathogens and plays vital roles in cell survival and homeostasis maintenance.^31^^,^^32^ The ATM–Chk2–P53 pathway was previously shown to participate in the maintenance of homeostasis in BRCA-1 deficient cells. During the embryonic development phase, the ATM–Chk2–P53 pathway eliminates mutant individuals by activating apoptosis, whereas the absence or low expression of Chk2 prevents the elimination effect. In addition, the ATM–Chk2–P53 pathway prevents tumorigenesis, and mutations in Chk2 and P53, which otherwise lead to the occurrence and proliferation of various tumors. Overactivation of the ATM–Chk2–P53 pathway may cause overactivation of apoptosis, which leads to cell senescence. In summary, these findings indicate that the ATM–Chk2–P53 pathway is vital for maintaining cellular homeostasis and a balance between tumor elimination and cell senescence.^33^

Previous research has focused on the maintenance of reactive oxygen species (ROS) homeostasis under oxidative stress. ROS levels can increase during long-term glucose starvation and oxygen deficiency, although excessive ROS production can lead to cell death. Previous findings have shown that the ATM–Chk2 pathway is activated by ROS elevation in a DNA-damage-independent manner, and Chk2 phosphorylates Beclin-1 at Ser90 and Ser93 site, and then blocks the inhibitory effects of Bcl-2 on Beclin-1. Activated Beclin-1 induces cell autophagy and eliminates injured mitochondria, which helps maintain ROS homeostasis.^34^ ULK1, a serine/threonine-protein kinase, is another vital factor for autophagy. AMPK phosphorylates ULK1 on Ser 317 and 777, which activates ULK1 and promotes autophagy during starvation, whereas mTOR phosphorylates Ser 757 and impairs Ser 317 and 777 phosphorylation, resulting in inhibited autophagy in the presence of a sufficient nutrition supply. Thus, the regulation of ULK1 phosphorylation is important in autophagy. Stress-induced ROS production activates the ATM–Chk2 pathway, and phosphorylated Chk2 phosphorylates ULK1 at Ser556, which promotes autophagy progression. In summary, the above findings indicate that the ATM–Chk2–ULK1 axis initiates autophagy to maintain ROS homeostasis and inhibit stress-induced apoptosis.^35^^,^^36^

**Figure 3:**
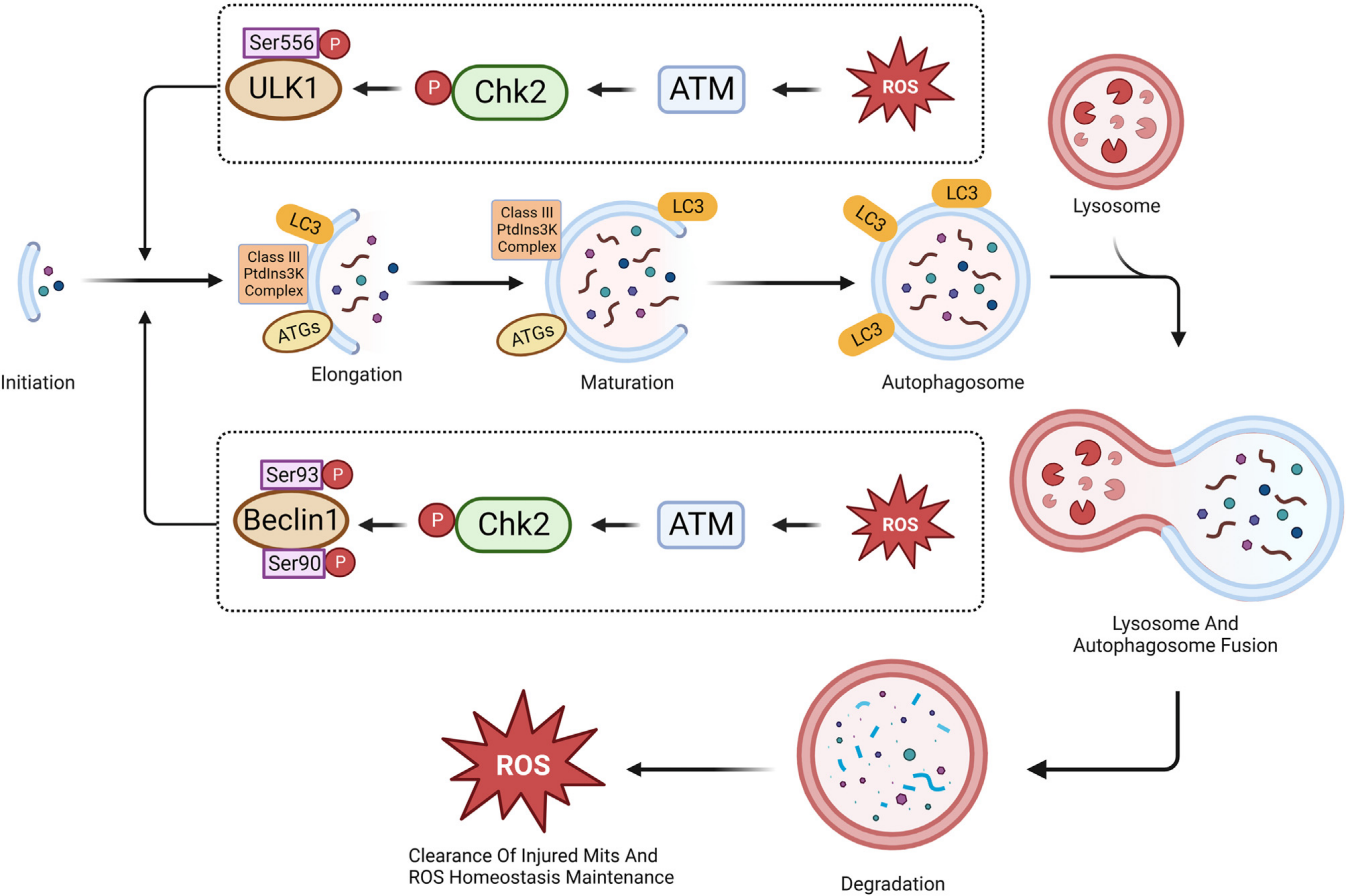
Chk2 maintains ROS homeostasis by regulating autophagy pathways. ROS accumulation induces cell autophagy, and excessive ROS activates the ATM-Chk2 pathway, leading to the phosphorylation of Chk2. The phosphorylated Chk2 in turn phosphorylates ULK1 at Ser556 and Beclin1 at Ser90 and Ser93, respectively, which are crucial factors in the early stage of autophagosome formation. Subsequently, through processes such as elongation, maturation, and lysosome-autophagosome fusion, Chk2 eliminates damaged mitochondria and ultimately maintains ROS homeostasis in the cytoplasm.

Autophagy following DNA damage can be promoted by Chk2 after it interacts with Foxk1 and Foxk2. Once phosphorylated, Chk2 can phosphorylate Foxk1 on S130 and Foxk2 on S61, which induces Foxk migration from the nucleus to the cytoplasm. 14-3-3γ binds with Foxk and sequesters it in the cytoplasm. Foxk migration weakened its ability to inhibit the expression of autophagy-related genes, which promoted autophagy. It was also confirmed that ATM-dependent phosphorylation of Chk2 at threonine 68 was essential for the interaction between Chk2 and Foxk. ATM and Chk2 inhibition also decreased autophagy caused by DNA damage.^37^ Microcystin-LR, a kind of intracellular toxin, caused DNA damage in male germ cells mediated by oxidative stress. Autophagosomes and the up-regulation of autophagy-related proteins were both observed in microcystin-LR-treated cells, which was reversed using the ATM inhibitor KU55933. These outcomes show that microcystin-LR-dependent ATM–P53 pathway activation may promote autophagy^38^ (Fig. 3).

## Chk2 mutations and expression levels in digestive-system tumors

### Influence of Chk2 mutations in digestive-system tumorigenesis

Mutations in the CHEK2 gene, which encodes Chk2, has been shown to increase the risk of developing a few different cancers.^39^ The founder CHEK2 mutation c.1100delC is a moderate-risk variant in breast cancer, whereas the p.I157T is a low-risk variant.^40^ The c.1100delC mutation can also increase the risk for mortality due to melanoma and prostate cancer^41^ In addition, germline CHEK2 mutations have been associated with prostate and breast cancer genesis. A series of myeloid malignancies, including myelodysplastic syndromes and chronic lymphocytic leukemia, have also associated with germline CHEK2 mutations.^42^^,^^43^

### Chk2 mutations in CRC and ESC

In a study involving Dutch participants, the c.1100delC mutation was detected in 10 of 237 (4.2 %) patients with hereditary nonpolyposis colorectal cancer and related diseases, which is four-fold higher than that in general Dutch population. Conversely, the results of a separate study showed that only 2 % of English patients with CRC carried the c.1100delC mutation. The frequency of Chk2 mutations is low in patients with CRC, and c.1100delC makes few contributions to colorectal tumorigenesis.^44^

Similar outcomes were observed in a study of ESC. The c.1100delC mutation was found in 0.5%, 1.5%, 3.0%, or 1.5% of patients with squamous cell carcinomas, adenocarcinomas, Barrett esophagus, or dysplasia, respectively, a prevalence rate that was not significant different with that among control individuals (1.4%). Thus, the c.1100delC mutation does not obviously promote ESC tumorigenesis.^45^ R145W, the only Chk2 missense mutation among 56 cell lines identified in previous research, cannot be phosphorylated by ATM and may promote tumorigenesis by decreasing Chk2 expression. Another missense mutant of Chk2, I157T, was found to increase the risk of CRC in both Poland and Finnish patients. Interestingly, this mutation may not cause the hereditary risk for CRC. In addition, no CHEK2-truncation mutations have been shown to increase the risk for CRC in Poland patients, although the carrier rate among Poland patients with CRC is 1.0%.^46^, ^47^, ^48^

### Chk2 mutations in GC and hepatocellular cancer (HCC)

The results of a Polish population-based study showed that CHEK2 mutations were present in 57 of 658 patients with GC, where truncating mutations and the missense mutation I157T accounted for 13 and 44 cases, respectively. Both mutations increase the risk of developing GC, and the truncation mutation seems to have a stronger effect than the missense mutation.^49^ In addition, germline Chk2 mutations do not lead to familial aggregation of GC,^50^ similar to previous findings for CRC. Interestingly, in a study focused on Turkish patients with HCC, the I157T and c.1100delC mutants were not detected in any of the 611 samples studied, including 165 patients with HCC and 446 individuals without any hepatic diseases. The I157T and c.1100delC mutants do not appear to increase the risk for HCC in the Turkish population. Similarly, among Chinese patients with HCC, ATM and BRCA2 are the most frequently mutated genes among genes related to the DDR pathway. In summary, the prevailing evidence does not indicate that CHEK2 mutations promote HCC tumorigenesis.^51^^,^^52^

### Chk2 mutations in pancreatic cancer

In a familial study, Bartsch et al selected 35 families with at least two members who had developed pancreatic cancer. The c1100 mutation was detected in only one of these families, in which two individuals were diagnosed with pancreatic cancer. Similarly, the c.1100delC and del5395 mutations, as well as I157T (the most common mutation in the study), were found to make little contribution to the mortality of patients of Czech Caucasian ancestry with sporadic pancreatic cancer. Thus, the c.1100delC may not increase the morbidity of patients with familial or sporadic pancreatic cancers.^53^, ^54^, ^55^ However, in recent studies, founder mutations in CHEK2 (including 1100delC, IVS2+1G > A, del5395, and I157T) were found to increase the risk of familial pancreatic ductal cancer, especially the I157T mutant. The I157T mutation was also shown to increase the risk of sporadic pancreatic ductal adenocarcinoma in another study.^56^, ^57^, ^58^ We hypothesize that the adverse outcomes may have been due to the different study groups, races, and DNA-sequencing methods involved, not only for pancreatic cancers, but also for other digestive-system cancers. In summary, it is difficult to explore the entire spectrum of Chk2 mutations that influence digestive-system tumorigenesis because they appear to be race-specific. More and larger studies should be conducted to explore this topic further.

### Abnormal Chk2 expression levels and digestive-system tumorigenesis

Chk2 expression is up-regulated in GCs with P53 mutations. Cells increase upstream Chk2 expression as a compensatory response to downstream P53 mutations.^59^ The outcomes of two studies focused on the relationship between Chk2 and GC prognosis indicated that Chk2 may participate in GC tumorigenesis as a cell-cycle regulator. Chk2 loss was documented in 14.1% of patients in the training group and 12.2% of patients in the validation group. Chk2 loss is an independent prognostic indicator of a poor prognosis, whereas Chk2 positivity is strongly related to a longer disease-specific survival time.^60^^,^^61^ In a recent study, Chk2 levels were clearly lower in GC tissues than in normal tissues, whereas phosphorylated ATM and Chk2 were clearly higher in advanced GCs. We speculate that Chk2 serves as a barrier for early GC development, whereas the loss of Chk2 protein expression may cause tumor progression. Interestingly, the results of that study also indicated that Chk2 expression may not significantly affect GC prognosis.^62^ In contrast, ATM and Chk2 seemed to be overexpressed in GC tissues that lacked ARID1A expression, which is mutated in about 30% of all GCs. The overexpression may reflect a compensatory mechanism due to impairment of the ATR–Chk1 axis.^63^

Stawinska et al found that Chk2 expression levels were lower in nearly half of CRCs studied, particularly those in stage A or B (according to the Dukes classification scheme). Approximately 30% of CRCs were observed to have up-regulated Chk2 expression. Previous results showed that Chk2 expression levels may depend on the degree of DNA damage and that higher Chk2 levels may promote tumorigenesis at an early stage.^64^ Chk2 overexpression was also observed in *Fusobacterium nucleatum-*related CRC. Interestingly, Chk2 expression in CRCs differs from that in GCs. Chk2 overexpression can specifically promote *Fusobacterium nucleatum*-related CRC progression. FadA, a newly characterized adhensin, can enhance E-cadherin and β catenin activation, which up-regulates Chk2 expression and finally causes Chk2 mediated DNA damage, whereas FadA knockdown can decrease Chk2 expression.^65^ Aljarbou et al presented evidence indicated that Chk2 expression was related to the location of CRCs, where higher Chk2 expression was observed in rectal cancer tissues and slightly lower Chk2 expression was observed in colon cancer tissues.^66^^,^^67^

### Chk2 and the radiochemotherapy sensitivities of digestive-system tumors

Radiochemotherapy is a traditional cancer-treatment method that involves the induction of tumor cell DNA damage, which triggers a series of downstream DDRs. Cell-cycle arrest and effective DNA repair can induce resistance to radiation and chemotherapy. Thus, targeting proteins related to DNA repair may lead to the untimely repair of radiochemotherapy-induced DNA damage and subsequent tumor cell death. Chk2 is known to play important roles in the survival of tumor cells and previous findings have demonstrated that it may decrease the effectiveness of DNA-targeting chemotherapy drugs. Inhibiting the DDR pathway and checkpoint events can improve the therapeutic effect of conventional genotoxic drugs.^68^^,^^69^

### Gastric cancer

Circular RNAs play important roles in tumorigenesis and tumor cell invasion by competitively binding to microRNAs that promote tumor cell proliferation. Hsa_circ_0001546, a type of circular RNA, was down-regulated in GCs and was related with a poor prognosis. Hsa_circ_0001546 can bind to miR-421 and thereby reverse mir-421-induced ATM down-regulation and tumor progression. Hsa_circ_0001546 overexpression was observed in GC cell lines pretreated with oxaliplatin, which up-regulated the expression and activation of ATM, Chk2, and P53. Oxaliplatin sensitivity in GCs could be increased by inhibiting proliferation and activating apoptosis-related pathways.^70^^,^^71^ Unconventional prefoldin RPB5 interactor (URI) was reported to promote cancer progression in previous studies. Bian et al reported that URI decreased cisplatin-induced DNA damage and apoptosis, which led to cisplatin resistance. In URI-knockdown GC cells, the phosphorylation levels of ATM and Chk2 were significantly decreased. These findings serve as a reminder that URI silencing increases the sensitivity of GCs to cisplatin by inhibiting the ATM–Chk2 pathway, which inhibits DNA repair.^72^

### Colorectal cancer

Hsieh et al found that Chk2 phosphorylation increases in oxaliplatin-resistant colorectal cells, suggesting that a high level of DNA repair occurs. Poly-adenosine diphosphate ribose polymerase 1 is another important factor that is important for DNA repair, and its interaction with Chk2 can promote DNA repair further. Interestingly, oxaliplatin therapy enhanced this process, which meant that the self-repair function of tumor cells also increased, which led to oxaliplatin resistance.^73^ However, the results of another two studies on 53BP1 showed that chemotherapy sensitivity can be regulated through the ATM–Chk2–P53 pathway. 53BP1 knockdown in cells treated with 5-fluorouracil can down-regulate Chk2 and pro-apoptotic proteins, which promotes tumor cell proliferation and leads to 5-fluorouracil tolerance. The results of another study indicated that icotinib hydrochloride treatment could increase the expression levels of apoptosis-related proteins (ATM, Chk2, and P53), resulting in a radiosensitizing effect. 53BP1 knockdown inhibited apoptosis induced by icotinib hydrochloride and the expression of proteins in ATM pathways, while inducing tumor proliferation.^74^^,^^75^ Thus, activating the ATM pathway can result in opposite effects in different experimental settings, and more research is needed to explore the related mechanisms.

4′-demethyldeoxypodophyllotoxin glucoside (4DPG) inhibits CRC progression and relieves chemotherapy resistance through a unique mechanism. In fluorouracil-resistant CRC cells, vimentin overexpression and the epithelial–mesenchymal transition were observed, which promoted tumor invasion and metastasis. 4DPG up-regulated Chk2 expression and phosphorylation, which inhibited the epithelial–mesenchymal transition and vimentin expression and phosphorylation. Thus, 4DPG inhibited fluorouracil-resistant CRC invasion and metastasis, indicating that 4DPG may contribute to chemotherapy resistance in CRC.^76^, ^77^, ^78^ The results of a study on radiotherapy resistance showed that the X-ray radiation resistance associated 1 gene may promote CRC progression through ATM–Chk1/2-mediated pathways, and its low expression enhanced the phosphorylation levels of ATM, Chk1 and Chk2, which promoted tumor cell-DNA repair and led to radioresistance.^79^

### Esophageal cancer

Apatinib is a tyrosine kinase inhibitor that was shown to enhance radiosensitivity in ESC. Pretreatment with apatinib increased G2/M arrest induced by radiotherapy, which may result from the down-regulation of Chk2 phosphorylation induced by apatinib. Thus, apatinib inhibits the DNA-repair process in tumor cells and further enhances radiosensitivity.^80^^,^^81^ In a mouse model of ESC, Chk1 and Chk2 expression increased significantly in 53BP1-knockdown cells. P53 down-regulation induced by 53BP1 knockdown also up-regulated Chk1 and Chk2 expression in mouse models. In summary, 53BP1 knockdown can significantly decrease the radiosensitivity of ESC.^82^ This conclusion differs from that reached in studies of 53BP1 and the Chk2 pathway in CRCs, which may reflect tissue specificity. TAB182 up-regulation was observed in ESC cells, which led to a poor prognosis after radiotherapy. TAB182 might induce radioresistance by influencing the Chk2–CDC25C signaling pathway. TAB182 acts as a downstream effector of Chk2 in the Chk2–CDC25C signaling pathway, leading to CDC25C phosphorylation and G2/M phase arrest. Down-regulating TAB182 leads to decreased CDC25C phosphorylation and inhibits G2/M arrest, which leads to ESC cell apoptosis and mitotic catastrophe. Thus, down-regulating TAB182 can enhance radiosensitivity in ESC.^83^

### Other digestive-system cancers

c-Rel is essential for effective DNA repair after genotoxic injury occurs in HCC because Chk2-mediated DNA repair is inhibited in c-Rel-deficient hepatic cells. C-Rel regulates the ATM–Chk2–P53 pathway and thereby limits DNA damage caused by genotoxicity and maintains genome stability and cell survival. The c-Rel inhibitor IT-603 can be used to increase the sensitivity of doxorubicin therapy.^84^^,^^85^ Cisplatin has been found to induce Chk1/2 phosphorylation and induce G2/M phase arrest. In lenvatinib-resistant HCCs, Chk1/2 might be crucial for maintaining cisplatin sensitivity.^86^ Histone chaperone anti-silencing function 1B (ASF1B) has been proved to be a diagnosis and prognosis biomarker of cancers and its up-regulation is related with poor prognosis. ASF1B down-regulation enhanced DDR-induced cell-cycle arrest in pancreatic cancer cells, which may be caused by Chk2 overexpression and phosphorylation. In cisplatin-treated cells with down-regulated ASF1B expression, late-apoptotic cells and apoptosis-related protein levels were both higher than in untreated cells. These findings indicate that ASF1B enhances cisplatin sensitivity by promoting apoptosis. However, the results of that study did not clarify whether apoptosis was a downstream effect of Chk2 pathway activation. Thus, the relationship between Chk2 activation and cisplatin resistance of pancreatic cancer cells should be investigated further.^87^^,^^88^

## Chk2 inhibitors and agents developed to target Chk2

### Chk2 inhibitors

Chk2 is overexpressed in some kind of cancers; thus, high Chk2 expression may be essential for DNA repair and tumor cell survival. In contrast to other components of the DDR pathway, Chk2 is recognized as an effective target for enhancing the efficacy of genotoxic treatments. This type of therapy inhibits the DDR pathway by blocking DNA repair and cell-cycle arrest or by preferentially activating senescence or mitotic-mutation programs in tumor cells. In addition, Chk2 inhibition may lead to a robust apoptosis response in tumor cells and protect normal tissues during radiochemotherapy.

The nonselective checkpoint kinase inhibitor, AZD7762, can enhance the sensitivity to cisplatin in ovarian clear cell carcinoma and the sensitivity to gemcitabine in urothelial carcinoma.^89^^,^^90^ Acetyl-macrocalin B (A-macB) inhibits tumor cell proliferation and induces apoptosis through the ROS–P38 MAPK signaling pathway. A-macB also causes DNA damage and activates the ATM–Chk2 pathway to induce G2/M phase arrest and DNA repair. AZD7762 abrogates A-macB-induced DNA repair and enhances cell cytotoxicity, whereas A-macB enhances AZD7762-depedent Chk2 inhibition. The results of that study indicated that a synergistic effect may occur between AZD7762 and A-macB and that combination treatment with both agents may lead to a better effect.^91^ However, adverse reactions, such as increased troponin expression and chest pain, were observed in patients treated with AZD7762. Studies on AZD7762 were halted because of the unpredictable cardiac toxicity.^92^ NSC109555, a selective Chk2 inhibitor, inhibited the phosphorylation of Chk2 induced by gemcitabine in pancreatic adenocarcinoma cells. In addition, NSC109555 enhanced the ROS-dependent gemcitabine-mediated tumor cell apoptosis.^93^ Pyrazole–benzimidazole conjugates are novel Chk2 inhibitors found to enhance the chemotherapy effects of cisplatin and doxorubicin. Galal et al developed various pyrazole–benzimidazole conjugates and evaluated their ability to inhibit Chk2 activation. The results of previous studies demonstrated that all the novel compounds could inhibit Chk2 and that compounds 10a-c, 14, and 15 enhanced the genotoxicities of cisplatin and doxorubicin, whereas compounds 9b and 20e23 decreased their genotoxicities.^94^, ^95^, ^96^

### Agents that target Chk2-related pathways

As a key factor in cell-cycle progression and cell homeostasis, regulating the Chk2 pathway may affect tumor cell survival; thus, efforts have been made to develop various compounds that can inhibit tumor cell growth by regulating the Chk2 pathway. The ATM–Chk2–P53 pathway is a vital part for α-pinene-induced G2/M phase arrest in HCCs, and activation of the ATM–Chk2–P53 pathway is associated with amentoflavone-induced CRC inhibition *in vitro* and *in vivo*. The cytotoxicity of IMB5043 in various tumor cells also involved the Chk2-mediated G2/M phase cell-cycle arrest and downstream apoptosis induction.^97^, ^98^, ^99^ A novel therapeutic method aimed at targeting Chk2-null CRC cells was developed by Ahmad et al. The peroxiredoxin-2 (PRDX2) enzyme inhibitor, N-carbamoyl alanine (NCA), was carried by nanoparticles to deliver drugs to tumor tissues more efficiently. PRDX2 is an important regulatory factor that decreases intracellular ROS levels. Because of the inhibition of PRDX2 and lost Chk2 expression, DNA double-strand breaks induced by ROS increase when DNA-repair function is deficient. The sustained DNA damage and accumulation of errors during faulty DNA repair can ultimately lead to tumor cell death.^100^

### Agents that target ATM

Most previous studies have focused on the radiochemosensitizing role of ATM inhibition, including exogenous ATM inhibitors and intrinsic ATM deficiency.^27^ Ku 55933 was the first selective ATM inhibitor developed. Ku 55933 showed a radiosensitizing effect by inhibiting the phosphorylation of downstream molecules mediated by ATM while enhancing the sensitivity to double-strand break-induced chemotherapeutics.^101^ Subsequently, Ku 60019 and Ku 59403 were developed as improved inhibitors that showed greater bioavailabilities. AZD 0156 and AZD 1390 are recently developed orally ATM inhibitors with a higher selectivity and greater ability to penetrate the blood–brain barrier.^102^ Currently, three ATM inhibitors are being tested in clinical trials; M4076, AZD1390, and Ku 60019 target advanced solid tumors, glioblastoma, and kidney cancer, respectively. It is worth mentioning that Ku 60019 will be combined with protein kinase CK2 inhibitors to explore a more effective treatment for kidney cancer in a prospective clinical trial.^103^ In addition, ATM deficiency may be related to higher sensitivity to radiochemotherapy. Furthermore, PARP inhibitors were found to be more effective in patients with ATM-deficient metastatic or recurrent GC.^104^ These outcomes may have been caused by the synthetic-lethality effect in the DDR pathway. In summary, the effects observed when using ATM inhibitors alone are not satisfactory. Combining ATM inhibitors with other cytotoxic chemotherapeutic drugs, radiation, or other DDR inhibitors (such as PARP inhibitors) is a more effective approach.^102^

### Agents that target P53

P53 is a tumor suppressor that promotes cell-cycle arrest, senescence, and apoptosis to inhibit tumorigenesis and tumor cell proliferation. P53 activation may be a potent anti-tumor method, especially in P53-mutant cancers. Chrysin causes cytotoxicity by activating P53 and promoting P21 binding in an ATM- and Chk2-dependent manner in the absence of DNA damage. MANIO, a P53 activator, binds to the DNA-binding domain of P53 to enhance its transcriptional activity and can inhibit CRC cell growth. Similarly, in an ESC model, costunolide up-regulated P53 and P21 expression, which induced G1/S phase arrest and apoptosis.^105^, ^106^, ^107^ Reticulon-3 is an endoplasmic reticulum-resident protein that is essential for maintaining the function of the endoplasmic reticulum. Recently data showed that Reticulon-3 can suppress HCC genesis by activating the Chk2–P53 pathway through a unique mechanism. Reticulon-3 recruited Chk2 to the endoplasmic reticulum and activated it in a calcium-dependent manner; however, no DNA damage occurred during the activation process. P53 phosphorylation at Ser392, mediated by Chk2 activation, inhibited HCC proliferation and induced apoptosis.^108^ Nag et al found that auranofin could inhibit tumor growth by directly activating P53 pathways and that it could protect normal colorectal epithelium tissue from radiation injury by regulating P53-related pathways. Auranofin pretreatment inhibits apoptosis mediated by radiation-induced P53 acetylation and activates the downstream P21 protein to induce cell-cycle arrest, which finally led to the regeneration of epithelium tissue and decreased mitotic catastrophe.^109^ Reactivation of mutant P53 proteins may be another potent method for treating cancers with P53 mutations, considering that malfunctional P53 is common in various cancers. APR-246 and COTI-2 are P53 reactivators that have progressed to the clinical-trial phase and have demonstrated anti-tumor efficiency in preclinical models. More studies are needed to verify the feasibility of this mutation-reversion therapy method^110^ (Table 1).

**Table 1:** Agents that target Chk2 and related molecules.

Agent type	Regulatory mechanism(s)	Agent name(s)	Tumor type(s)	Clinical application(s)
Chk2 inhibitor	Inhibition of tumor cell-cycle arrest and DNA repair	AZD7762	Ovarian clear cell carcinoma; urothelial carcinoma	Increasing the sensitivity of cisplatin in ovarian clear cell carcinoma and the sensitivity of gemcitabine in urothelial carcinoma
Chk2 inhibitor	Inhibition of Chk2 phosphorylation and enhanced reactive oxygen species (ROS) dependent apoptosis	NSC109555	Pancreatic adenocarcinoma	Increasing the sensitivity of gemcitabine in pancreatic adenocarcinoma
Chk2 inhibitors		Pyrazole-benzimidazole conjugates	Breast cancer	Enhancing the efficiency of cisplatin and doxorubicin
An *ent*-diterpenoid isolated from *Isodon silvatica*	Induction of G2/M phase arrest and promotion of apoptosis	A-macB	Esophageal squamous cell carcinoma	Exhibiting synergistic anti-cancer effects with AZD7762
Natural compound isolated from pine needles	Activating the ATM–Chk2–P53 pathway	α-pinene	Hepatocellular carcinoma	Anti-tumor effect
Natural compound isolated from medicinal plants		Amentoflavone	Colorectal cancer	
A thiophenylated pyridazinone		IMB5043	Various cancers, especially hepatocellular carcinoma	
Peroxiredoxin-2 inhibitor	Inhibiting peroxiredoxin-2-mediated ROS clearance	NCA	Chk2-null colorectal cancer	
The first developed selective ATM inhibitor		Ku 55933		Increasing radiochemosensitivity
Improved derivatives of Ku 55933	ROS	Ku 60019 and Ku 59403		Increased bioavailability
ATM inhibitors		AZD 0156 and AZD 1390		Enhanced selectivity and a greater ability to penetrate the blood–brain barrier
ATM inhibitors in clinical trials		M4076	Advanced solid tumors	
		AZD1390	Glioblastoma	
		Ku 60019	Kidney cancer	Combined with protein kinase CK2 inhibitors to treat kidney cancer
P53 activators	P53 activation and the promotion of P53 binding to P21	Chrysin		Anti-tumor effect
	Interaction with the P53 DNA-binding domain and enhanced P53 transcriptional activity	MANIO	Colorectal cancer	
	P53 and P21 up-regulation and induced G1/S phase arrest and apoptosis	Costunolide	Esophageal cancer	
Endoplasmic reticulum-resident proteins	Activation of the Chk2–P53 pathway	Reticulon-3	Hepatocellular carcinoma	
A gold-containing compound	Inhibiting radiation-induced P53-acetylation-mediated apoptosis and P21 activation to induce cell-cycle arrest	Auranofin	Colorectal cancer	Protecting normal colorectal epithelium tissue from radiation-induced injury
P53 reactivators in clinical trials	Reactivation of mutant P53 proteins	APR-246 and COTI-2		Anti-tumor effects

## Perspectives and conclusions

Digestive-system cancers have high incidence rates and pose severe threats to human health. Although traditional genotoxic radiochemotherapies have prolonged survival times, radiochemotherapy resistance can occur. Thus, novel targets involved in tumorigenesis and invasion need to be identified in order to develop new anti-tumor treatments. Chk2 plays important roles in the DDR pathway regulating cell-cycle progression. Chk2 is also associated with tumorigenesis, tumor progression, tumor recurrence, drug resistance, and the prognosis of tumors, indicating that it is potentially an important therapeutic anti-tumor target. Currently, an increasing number of Chk2 inhibitors are being developed and explored in preclinical situations. The results of previous studies have confirmed that Chk2 expression and the application of Chk2 inhibitors can affect the reversal of drug resistance in digestive-system tumors; however, whether they can directly inhibit the occurrence and progression of tumors by regulating Chk2 alone remains unclear. The efficacy of combination treatment with Chk2 inhibitors and chemotherapeutic agents or S and G2/M phase checkpoint inhibitors in reversing digestive-system tumor resistance remains unexplored. Although Chk2 inhibitors show good prospects for increasing the sensitivity to chemotherapy, the stability and safety of some inhibitors need to be further demonstrated. In the future, an in-depth understanding of the roles of Chk2 in drug resistance of digestive-system tumors is expected to improve therapeutic effects and improve the survival and prognosis of patients with digestive-system tumors.

In addition, Chk2 mutations and abnormal Chk2 expression may be critical for digestive-system tumorigenesis. Founder mutations in Chk2, such as c.1100delC and I157T, seem to be the most studied variants. However, whether such mutations increase the risks for developing digestive-system cancers remains inconclusive. For example, previous findings conflict with each other in different studies involving different races. We can only conclude that variants such as c.1100delC and I157T may increase the risk of specific cancers in specific races. However, only a few studies have been focused on the entire coding sequence of CHEK2, and more studies are needed to reveal the overall situation for Chk2 in terms of the entire sequence across all races.

The reactivation of mutant P53 may be a viable method for treating cancers with P53 mutations. The reactivation of functionally deficient CHEK2 could be an effective method for treating cancers related to CHEK2 mutations. We speculate that detecting CHEK2 mutations might be helpful for predicting the risks for developing specific cancers. In addition, Chk2 expression varies in different tumors. We hypothesize that Chk2 expression is tissue-specific or even individualized, which make it difficult to develop anti-tumor methods that target Chk2. The expression level of Chk2 could be related to the neoplastic stage. For early-stage tumors, Chk2 may be highly expressed and serve a protective role, and as Chk2 expression is gradually lost, a tumor might progress to an advanced stage. Thus, the Chk2 level might be vital for determining the prognosis of specific tumors.

Autophagy and homeostasis maintenance are both essential for cell survival and normal cellular functions. Chk2 participates in autophagy by phosphorylating Foxk and ULK1, which activates the ATM–Chk2–ULK1–Foxk axis. In addition, Chk2 participates in maintaining homeostasis under oxidative stress by phosphorylating Beclin1 and ULK1, which inhibits ROS production and promotes mitochondrial autophagy in injured cells. However, autophagy activation may inhibit stress-induced apoptosis, which may obstruct tumor cell death. The results of previous studies have shown that chemotherapy resistance may be caused by high activation of autophagy, which can be reversed by a combination of autophagy inhibitors and chemotherapeutic agents. In summary, Chk2-mediated autophagy pathways may represent novel targets for overcoming chemotherapy resistance.

In conclusion, Chk2 is vital for cell-cycle arrest and the DDR pathway. Chk2 also participates in autophagy pathways and maintaining cellular homeostasis. Chk2 mutations can lead to failed DNA repair and the accumulation of mistakes during mitosis, resulting in tumorigenesis. Some Chk2 variants have been shown to increase the risks for various cancers, although inconsistent outcomes have been reported. Newly developed agents targeting Chk2 and other related molecules may reverse radiochemotherapy resistance and inhibit tumor progression by regulating Chk2 pathways. However, Chk2 up-regulation or down-regulation may depend on the tumor type, tumor stage, or even the individual genetic background. In summary, studies of Chk2 have brought hope for treating digestive-system cancers.

## Author contributions

Conceptualization and supervision: F.Y. and C.W.; writing—original draft: Y.A. and D.G.; writing—review and editing: Y.H., N.G., and J.G.

## Conflict of interests

The authors declare no conflict of interests.

## Funding

The study was supported by the 10.13039/501100001809National Natural Science Foundation of China (No. 82100700 to F.Y.), the 10.13039/501100002858China Postdoctoral Science Foundation (No. 2020M670101ZX to F.Y.), the Fundamental Scientific Research Project from the 10.13039/501100007620Educational Department of Liaoning Province, China (No. LJKMZ20221191 to F.Y.), and the 345 Talent Project of Shengjing Hospital of China Medical University (No. 52-30B to F.Y.).
